# *Pantoea* sp. P37 as a novel nonpathogenic host for the heterologous production of rhamnolipids

**DOI:** 10.1002/mbo3.1019

**Published:** 2020-02-29

**Authors:** Margarete Monika Nawrath, Christoph Ottenheim, Jin Chuan Wu, Wolfgang Zimmermann

**Affiliations:** ^1^ Department of Microbiology and Bioprocess Technology Institute of Biochemistry Leipzig University Leipzig Germany; ^2^ Institute of Chemical and Engineering Sciences Agency for Sciences, Technology and Research (A*STAR) Singapore City Singapore

**Keywords:** biosurfactants, genome sequencing, heterologous expression, *Pantoea*, rhamnolipids

## Abstract

Microbially derived surfactants, so‐called biosurfactants, have attracted significant attention as an environmentally friendly alternative to their chemically synthesized counterparts. Particularly, rhamnolipids offer a large potential with their outstanding surfactant properties such as complete biodegradability, low toxicity, and stability. Rhamnolipids are naturally synthesized by the opportunistic human pathogen *Pseudomonas aeruginosa* under the tight regulation of a highly complex quorum‐sensing system. The heterologous production of mono‐rhamnolipids by a newly isolated nonpathogenic strain of the genus Pantoea was investigated. Analysis of the genome obtained by a chimeric assembly of Nanopore long reads and high‐quality Illumina reads suggested that the strain has evolved to an epiphytic rather than a pathogenic lifestyle. Functional heterologous expression of the mono‐rhamnolipid operon rhlAB derived from a *P. aeruginosa* strain was established and confirmed by HPLC analysis. Transcriptome analysis indicated destabilizing effects of the produced rhamnolipids on the cell envelope of the host resulting in the induction of molecular stress responses. After integration of the rmlBCDA operon, extracellular rhamnolipids in amounts up to 0.4 g/L could be detected and were identified as a mono‐rhamnolipid Rha‐C_10_‐C_10_ by MALDI‐TOF mass spectrometry.

## Graphical abstract

Biosurfactants such as rhamnolipids are emerging as a sustainable and environmentally friendly alternative to synthetic surfactants. The production of the mono‐rhamnolipid Rha‐C10‐C10 by a novel nonpathogenic and easily engineerable host, *Pantoea* sp. P37 was investigated. Analysis of the genome suggested that the strain has evolved into an epiphytic rather than a pathogenic lifestyle. Functional heterologous expression of the mono‐rhamnolipid operon rhlAB derived from a *Pseudomonas aeruginosa* strain was established.

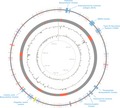

## 1 INTRODUCTION

Rhamnolipids are low molecular weight glycolipids exhibiting favorable physiochemical properties as biosurfactants, such as a high surface activity and low critical micelle concentrations. Their industrial applications are similar to those of other biosurfactants, ranging from biodegradable household cleaning products, cosmetics, food additives, environmental bioremediation, for enhanced oil recovery or as a nontoxic ingredient in pharmaceutical and medical products (Henkel et al., 2012). Rhamnolipids are composed of one (mono‐rhamnolipids) or two L‐rhamnose sugars (di‐rhamnolipids) bound to one or more activated β‐hydroxy fatty acids of varying chain length (C_8_‐C_16_) and degree of saturation. Typically, mixtures of rhamnolipid congeners are secreted by the producing microorganism depending on its phylogeny and the fermentation conditions (Abdel‐Mawgoud, Lépine, & Déziel, 2010). Two main reactions are involved in rhamnolipid biosynthesis: the dimerization of two β‐hydroxy fatty acids for the formation of the lipophilic moiety, and the transferase reaction for the glycosidic conjugation of the activated nucleotide carbohydrate dTDP‐L‐rhamnose to the fatty acid dimer. The dTDP‐L‐rhamnose moiety is formed by an enzymatic four‐step reaction encoded by the *rmlBCDA* operon. The dimerization step is catalyzed by the 3‐(3‐hydroxyalkanoyloxy)alkanoate synthetase, encoded by the *rhlA* gene, and the transferase reaction by the rhamnosyltransferase I, encoded by the *rhlB* gene. The genes *rhlA* and *rhlB* are organized in one operon. For the synthesis of a di‐rhamnolipid, a second transferase step is included, catalyzed by the rhamnosyltransferase II (RhlC) and encoded by the *rhlC* gene (Abdel‐Mawgoud, Hausmann, Lépine, Müller, & Déziel, 2011). Rhamnolipids are naturally produced by *Pseudomonas aeruginosa* together with various virulence factors during biofilm formation. While rhamnolipids are important structural integrity components in biofilms, they are also responsible for necrosis of host macrophages and leukocytes during acute infections (Lee & Zhang, 2015). The World Health Organisation recently published data underlining the emerging threat of antibiotic‐resistant *P. aeruginosa* strains (Tacconelli, Magrini, Kahlmeter, & Singh, 2017). Rhamnolipid biosynthesis is regulated by multilevel signal transduction networks such as the quorum‐sensing system which involves multiple regulatory factors, secondary messengers, and signaling molecules. Since the pathogenicity and complexity of the quorum‐sensing system in *P. aeruginosa* is restricting its use for the industrial‐scale production of rhamnolipids, many studies have focused on the discovery of alternative nonpathogenic microbial producers. The majorities of previously described strains were either members of the Pseudomonadaceae family such as *P. chlororaphis*, *P. fluorescens*, *P. lueola*, *P. indica*, and *P. stutzeri* or were part of the genus Burkholderia (Abdel‐Mawgoud et al., 2010). A maximum yield of naturally produced rhamnolipids of 9.6 g/L has been reported with a strain of *P. indica* (Bhardwaj, Cameotra, & Chopra, 2015). Another approach for the safe production of rhamnolipids is by heterologous recombination using a safe alternative host strain instead of *P. aeruginosa*. Besides the standard model organisms for genetic recombination *E. coli* and *S. cerevisiae*, Pseudomonadaceae have been predominantly used for the heterologous production of rhamnolipids with *Pseudomonas putida* KT2440 being the most prominent strain exhibiting the highest reported rhamnolipid titer of 14.9 g/L (Beuker et al., 2016). Although *P. putida* KT2440 has been declared as biologically safe by the US Recombinant DNA Advisory Committee, the strain contains a number of potential pathogenicity factors such as phospholipase D (Moore et al., 2006; Nelson et al., 2002).

*Pantoea* are Gram‐negative bacteria belonging to the Enterobacteriaceae family. Within the *Pantoea* genus, there are currently 21 known type strains (Walterson & Stavrinides, 2015). While most are assigned to BSL 1, a few species are considered animal or human pathogens (*P. agglomerans*, *P. brenneri*, *P. eucrina*, *P. septica*) and some are suspected plant pathogens (German Federal Institute for Occupational Safety and Health, 2015). However, some *P. agglomerans* BSL 2 strains are used commercially as biocontrol agents (strains C9‐1 and E325) and are considered as safe due to the lack of specific virulent determinants (Smits et al., 2010). So far, a total of 21 complete *Pantoea* genomes and 168 contigs and scaffolds of the 24 identified species have been deposited with the National Center for Biotechnology Information (NCBI). The majority (~80%) of the assemblies belong to only five assigned species providing limited genotypic insights into this diverse bacterial genus (NCBI Resource Coordinators et al., 2014).

The aim of this study was the identification of an alternative host for the heterologous production of rhamnolipids with a low safety risk profile and to uncouple the rhamnolipid synthesis from its complex quorum‐sensing system.

## 2 MATERIALS AND METHODS

### 2.1 Media, culture conditions, and strains

All strains were precultured in 25 ml Lennox broth (10 g/L, tryptone, 5 g/L yeast extract, 5 g/L NaCl) overnight at 37°C in shake‐flasks. As rhamnolipid production medium, 5 ml minimal salt medium M9 in 14‐ml Falcon tubes fortified with 0.4 g/L glycerol was used (0.7 g/L KH_2_PO_4_, 0.9 g/L Na_2_HPO_4_, 2 g/L NaNO_3_, 0.4 g/L MgSO_4_ · 7 H_2_O, 0.1 g/L CaCl_2_ · 2 H_2_O, 2 ml trace elements element solution: 2 g/L FeSO_4_ · 7 H_2_O, 1.5 g/L MnSO_4_ · H_2_O, 0.6 g/L (NH_4_)_6_Mo_7_O_24_ · 4 H_2_O). The production medium was incubated at 37°C under shaking conditions for 24 hr and for plasmid‐carrying strains, the medium contained 30 µg/L kanamycin. *Pseudomonas aeruginosa* TGR2A and *Pantoea* sp. P37 were isolated from soil samples, and *E. coli* OmniMAX was purchased from Thermo Fischer Scientific. *Pantoea* sp. P37 was deposited as DSM 32899 at the Leibniz Institute *DSMZ*‐German Collection of Microorganisms and Cell Cultures, Germany.

### 2.2 Screening for an alternative host for recombinant rhamnolipid production

Soil samples were mixed with sterile water. After a short sedimentation time, serial dilutions of the liquid phase were spread on selective LB agar plates which were fortified with 1 g/L R90 rhamnolipids in order to only promote the growth of strains resistant to rhamnolipids (AGAE Technologies). After incubation at 37°C overnight, single phenotypically distinguishable colonies were picked and inoculated into sterile LB broth fortified with 1 g/L R90 rhamnolipids, grown overnight and restreaked on the LB‐R90‐agar plate to assure isolation. Colony PCR was performed with the primer pair rhlI_F/rhlI_R targeting the rhamnolipid biosynthesis controlling acyl‐homoserine‐lactone synthase encoded by *rhlI* in *P. aeruginosa* genome. Isolates with a positive result, showing a 236‐bp amplicon on the agarose gel, were excluded. All other isolates were further identified with 16S rRNA sequencing using primer pair 27F/1492R.

### 2.3 Construction of a phylogenetic tree

The phylogenetic tree was constructed with MEGA6 based on the concatenated sequences of the three conserved genes *gyrB*, *rpoB,* and 16S rRNA (Kumar, Stecher, & Tamura, 2016). In an initial step, the genome assemblies from various *Pantoea* strains were downloaded from NCBI and the genes extracted (Table A2). The tree was rooted with the concatenated genes from *E. coli* K12. It was calculated based on the general time‐reversible model with a discrete gamma distribution (+G, parameter = 0.2441) and an allowance for evolutionary invariant sites (+I, 32.53% sites). All positions containing gaps and missing data were excluded from the analysis leading to a final of 9,599 positions in the final dataset. A bootstrap value of 1,000 replications was applied.

### 2.4 Genome sequencing and data analysis

The total genomic DNA from *Pantoea* sp. P37 was isolated following the protocol for Gram‐negative bacteria of the Promega Wizard Genomic DNA Purification Kit. To obtain high‐quality reads, genomic DNA was sequenced on an Illumina HiSeq 4000 platform (Illumina Inc.) yielding 150 bp PE short reads. Furthermore, genomic DNA was sequenced on an Oxford Nanopore MinIon (Oxford Nanopore Technologies) to obtain long reads for genome scaffolding. Short and long reads were assembled by Unicycler in a chimeric approach, and the resulting sequences yielding a complete genome of five circular sequences were annotated by Prokka (Seemann, 2014; Wick, Judd, Gorrie, & Holt, 2017). Geneious (Biomatters) was used for detailed DNA data analysis. Based on the virulence factor database (VFDB) of common bacterial pathogens, the VFanalyzer pipeline was used for the identification of virulence genes (Liu, Zheng, Jin, Chen, & Yang, 2018). IslandViewer 4 was used for the prediction of genomic islands. Prophage sequences were identified by PHASTER (Arndt et al., 2016; Bertelli et al., 2017; Zhou, Liang, Lynch, Dennis, & Wishart, 2011). Further annotations, indicating bacteriophage infections were analyzed manually. The bacterial version of antiSMASH was used to detect secondary metabolite biosynthetic gene clusters (Blin et al., 2019). The results were illustrated in a genome map created by the CGView Server and CIRCOS (Grant & Stothard, 2008; Krzywinski et al., 2009).

### 2.5 Construction of pETrhlAB

All enzymes used for restriction digestion reactions were purchased from New England Biolabs. The expression vector for heterologous mono‐rhamnolipid production was constructed following the Twin‐primer Assembly method (TPA) in combination with overlap extension PCR (Liang, Liu, Low, Ang, & Zhao, 2017). All final constructs were sequenced to guarantee the absence of mutations. The *rhlAB* operon derived from genomic DNA of *P. aeruginosa* TGR2A. *rhlA* was amplified with primer pairs rhlA_SF/rhlA_OER (Table A3) using Q5 polymerase (NEB). *rhlB* was amplified with primer pairs rhlAB_OEF/rhlB_SR using KOD Xtreme Hotstart (Merck). Both forward primers contained a synthetic RBS sequence designed with the Ribosome Binding Site Calculator tool with translation rates similar to the predicted natural ones of *P. aeruginosa* PAO1 (Salis, 2011). The two amplicons were subsequently fused via overlap extension PCR and used as a template for amplification with primers rhlA_SF/rhlB_LR and rhlA_LF/rhlB_SR in two separate reactions with KOD Xtreme Hot Start DNA Polymerase (Merck KGaA). The vector backbone part derived from pET28a(+) which was amplified with the primer pairs pET_SF/pET_LR and pET_LF/pET_SR. The final gel‐purified PCR products were assembled in one step together with the oligo pair J23108_F/J23108_R containing the constitutive promoter sequence obtained from BioBrick part BBa_J23108 (http://www.partsregistry.org). For one reaction, each fragment was diluted to reach a final concentration of 20 fmol per 20 µl. Two microlitre Cutsmart Buffer (New England Biolabs) was added to the mixture and topped up with nuclease‐free water. All fragments were assembled using the following time/temperature profile: 2 min at 98°C, 2 min at 85°C, 2 min at 75°C, 2 min at 65°C and 2 hr at 56.5°C (*T*_m_ of overlap regions + 5°C). Four microlitre of the ligation product mixture was transformed into Mix & Go! (Zymo Research) competent *E. coli* OmniMAX cells (Thermo Fisher Scientific) and grown overnight. Colonies were picked, plasmids extracted, and restriction digestion with XhoI and NheI‐HF performed. The correctly assembled construct, the expression vector pETrhlAB8, was finally transformed via electroporation into *Pantoea* sp. P37. For the construction of pETrhlAB100, the same fragments were used, only the oligo pairs were changed to J23100_F/J23100 carrying the promoter sequence of BBa_J23100.

### 2.6 Construction of pET3110 and pET2711

The construction of pET3110 and pET2711 started from the previously built vectors pETrhlAB8 and pETrhlAB100, respectively. The *rmlBCDA* operon derived from *P. aeruginosa* TGR2A and was subcloned into the vectors as one fragment by amplifying the region with primer pairs rml_SF/rml_LR and rml_LF/rml_SR. The purified PCR products were assembled together with fragments derived from amplification of the vectors pETrhlAB8 or pETrhlAB100 with primer pairs rhlvb_SF/ rhlvb_LR and rhlvb_LF/ rhlvb_SR. The construct was verified by restriction digestion with NotI‐HF.

### 2.7 Construction of plasmids pACYC_FAS, pACYC_fabH, pACYC_P450, and pACYC_algC

The backbone fragments of pACYC vectors were amplified with primer pairs pACYC_SF/pACYC_LR and pACYC_LF/pACYC_SR. All subcloned sequences were derived from genomic DNA of *P. aeruginosa* TGR2A. For pACYC_FAS, the genomic region containing genes *fabF1*, *fabG,* and *fabD* was amplified with fabDFG_SF/fabDFG_LR and fabDFG_LF/fabDFG_SR, the genomic region with *accA* was amplified with primers accA_SF/accA _LR and accA _LF/accA _SR, and *accD* with accD_SF/accD _LR and accD _LF/accD _SR. Genes *accB* and *accC* could be amplified as one fragment with accBC_SF/accBC _LR and accBC _LF/accBC _SR. The total amount of 10 fragments was mixed together after purification to a final concentration of 20 fmol of each part. The final hybridization step was performed at two temperatures, 1 hr at 56.2°C and 1 hr at 55.7°C. The extracted plasmids were examined by SmaI digestion.

For the construction of pACYC_fabH, primers fabH_SR/fabH_LR and fabH_SF/fabH_LF were used. The fragments were assembled at a hybridization temperature of 56°C and digested with BsgI for a final check. As the gene encoding the monooxygenase P450 is close to *fabH*, only one fragment needed to be subcloned to obtain the final construct pACYC_P450. Primers P450_SF/P450_LF and fabH_SR/fabH_LR were used to amplify the fragment from genomic DNA as a template. The hybridization temperature was 56°C and the assembly was verified by XhoI digestion. Through amplification with alg_LF/alg_SF and alg_SR/alg_LR, pACYC_algC was constructed with hybridization settings of 1 hr at 56.2°C and 1 hr at 55.7°C. The construct was verified by double digestion with SapI and XhoI.

### 2.8 Rhamnolipid quantification

Rhamnolipids in culture supernatants were quantified by HPLC/UV according to Smyth, Perfumo, Marchant, and Banat (2010) with some modifications using a Shimadzu LC‐20A HPLC with SPD‐20A UV/VIS detector (Shimadzu). Briefly, 100 µl of 1:10 water‐diluted, cell‐free supernatant was mixed with 100 µl derivatization reagent consisting of 0.5 M 2‐bromoaceto‐phenone: 1 M trimethylamine (8:2% v/v) in acetonitrile. The mixture was heated for 1 hr at 80ºC under constant shaking (1,000 rpm) and filtered with nylon syringe filters (0.22 mm). Gradient HPLC with UV detection set at 244 nm was used with a Phenomenex reversed‐phase column (C18 column, 250 mm × 4.6 mm × 5 mm i.d.), acetonitrile (mobile phase A), and phosphoric acid (mobile phase B, 3.3 mM). Gradient conditions were as follows: 50% A and 50% B for 3 min, 100% mobile phase A for 19 min and held for 5 min, 50% A over 3 min and finally held for 10 min. The flow rate was set at 1.0 ml/min, and the injection volume was 20 µl. Standard curves were created using commercial rhamnolipids (Sigma‐Aldrich).

### 2.9 Liquid–liquid extraction of rhamnolipids

The culture supernatant was filtered through a 0.22‐µm syringe filter, acidified with HCl to pH 2 and kept overnight at 4°C for rhamnolipid precipitation. The acidified supernatant was centrifuged at 25,000 *g* and 4°C for 10 min, and the pellet was dissolved in 4 ml water. Subsequently, 8 ml ethyl acetate was added, vortexed vigorously and centrifuged for 5 min for phase separation. The upper phase containing the rhamnolipids was transferred into a new tube, and the lower phase was extracted three more times with 8 ml ethyl acetate, respectively. After extraction, the ethyl acetate fraction was evaporated in a rotary evaporator at 40°C (Behrens, Engelen, Tiso, Blank, & Hayen, 2016).

### 2.10 MALDI‐TOF‐MS analysis of rhamnolipids

The ethyl acetate fraction containing the rhamnolipids was dissolved in 100 µl water. The matrix was prepared by mixing 20 mg of 2,5‐dihydroxybenzoic acid with 1 ml acetonitrile and agitated until complete dissolution. The matrix was mixed 1:1 with the sample. One microlitre of the obtained solution was spotted on a polished steel MALDI target plate and air‐dried before introduction and analysis in an autoflex maX MALDI system (Bruker) with a Smart Beam Laser in linear mode. Lens and reflector voltages were 33 and 3,133 V, respectively. For each spectrum, a number of 300 shots were acquired. The detector voltage was set to 2.5 GS/s. Signals smaller than 480 Da were suppressed (Price, Ray, Vermillion, & Kuo, 2009).

### 2.11 Transcriptome analysis by RNA sequencing

The recombinant *Pantoea* sp. P37 carrying the pETrhlAB8 vector and the *Pantoea* sp. P37 wild type (WT) were grown overnight at 37°C under shaking conditions (200 rpm) in glycerol‐fortified LB medium. The overnight cultures were centrifuged to pellet the cells. RNA sequencing was performed by Novogene (Singapore). For differential expression analysis, the readcount values were normalized by TMM (Trimmed Mean of M‐values). The DEGseq package was used for analysis (Wang, Feng, Wang, Wang, & Zhang, 2009). A volcano plot, plotting significance as −log 10 (*p*‐value) versus log2 fold change, was generated showing the overall distribution of differentially expressed genes. No biological replicates were included in the experimental setup; therefore, only outliers with high log2 fold changes and low p‐values were examined.

## 3 RESULTS

### 3.1 Isolation of a safe bacterial strain for heterologous production of rhamnolipids

The primary screening of soil samples on rhamnolipid‐fortified LB agar yielded 78 rhamnolipid‐resistant isolates. The exclusion of undesirable but ubiquitous *P. aeruginosa* strains by the amplicon‐positive/negative screening narrowed the pool further down to 9 candidates. Subsequent 16S rRNA sequencing revealed that all isolates belong to the Enterobacteriaceae family; however, 8 isolates were identified as members of the genera *Klebsiella*, *Enterobacter*, *Raoultella,* and *Cronobacter*, common BSL 2 microorganisms. One isolate was identified as a member of the genus *Pantoea* and designated as *Pantoea* sp. P37. *Pantoea* species are mostly classified as BSL 1. Its 16S rRNA sequence was deposited under accession number MH071150 at NCBI.

### 3.2 Genome analysis of *Pantoea* sp. P37

The genome of the isolate *Pantoea* sp. P37 was sequenced yielding high‐quality Illumina short reads and Nanopore long reads. The sequencing data were submitted to the NCBI Sequence Read Archive under accession numbers SRR7962492 and SRR7962491. The chimeric genome assembly yielded 5 circular elements with varying characteristics (Table A1). It consisted of one chromosome, two mega‐plasmids (>100 kb) and another two plasmids with a total genome size of 4,626,472 bp and an overall G + C content of 55.4%. The chromosome harbored a total number of 3,384 coding sequences (CDS), with 517, 252, 37, and 6 CDS for the corresponding plasmids which resulted in a total of 4,196 CDS for the entire genome. 8.2% of the chromosomal CDS were hypothetical protein CDS while 15.5% of the plasmidic CDS were unidentified. A total of 8 noncoding RNAs (ncRNA), 80 transfer RNAs (tRNA), and 22 ribosomal RNAs (rRNA), of which seven genes were 16S rRNAs, seven genes were 23S rRNAs, and eight genes were 5SRNAs. The *Pantoea* sp. P37 genome was deposited in the NCBI repository under accession number CP032702‐CP032706, BioProject ID PRJNA494309, biosample ID SAMN10160529.

### 3.3 Phylogenetic analysis and taxonomic affiliation

**Figure A1:**
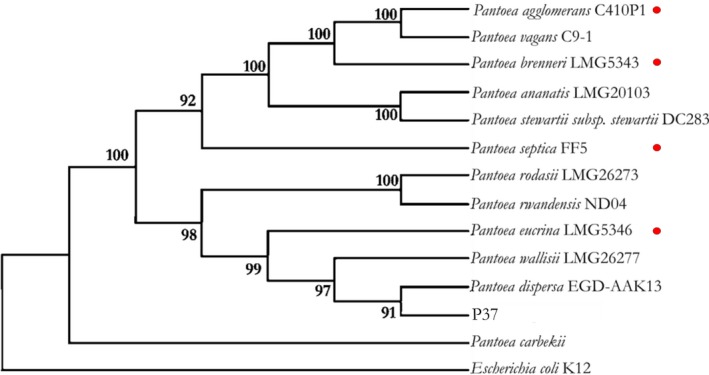
Maximum likelihood tree showing the phylogenetic relationship between *Pantoea* sp. P37 and selected *Pantoea* strains based on 16S rRNA, gyrB and rpoB. The percentage of trees in which the associated taxa clustered together is shown next to the branches. Red colored dots indicate a BSL2 classification according to the German Federal Institute for Occupational Safety and Health (2015)

For species classification, a maximum likelihood tree illustrating the phylogenetic relationship between *Pantoea* sp. P37 and 12 selected *Pantoea* strains was constructed (Figure A1). The isolated strain *Pantoea* sp. P37 showed only a loose evolutionary relationship with the virulent strains *P. agglomerans*, *P. brenneri*, *P. septica,* and *P. eucrina* within the displayed *Pantoea* entity. It exhibited a close relatedness to *P. dispersa* and *P. wallisii* which are both classified as BSL 1 strains.

### 3.4 Genome Mapping

**Figure 1:**
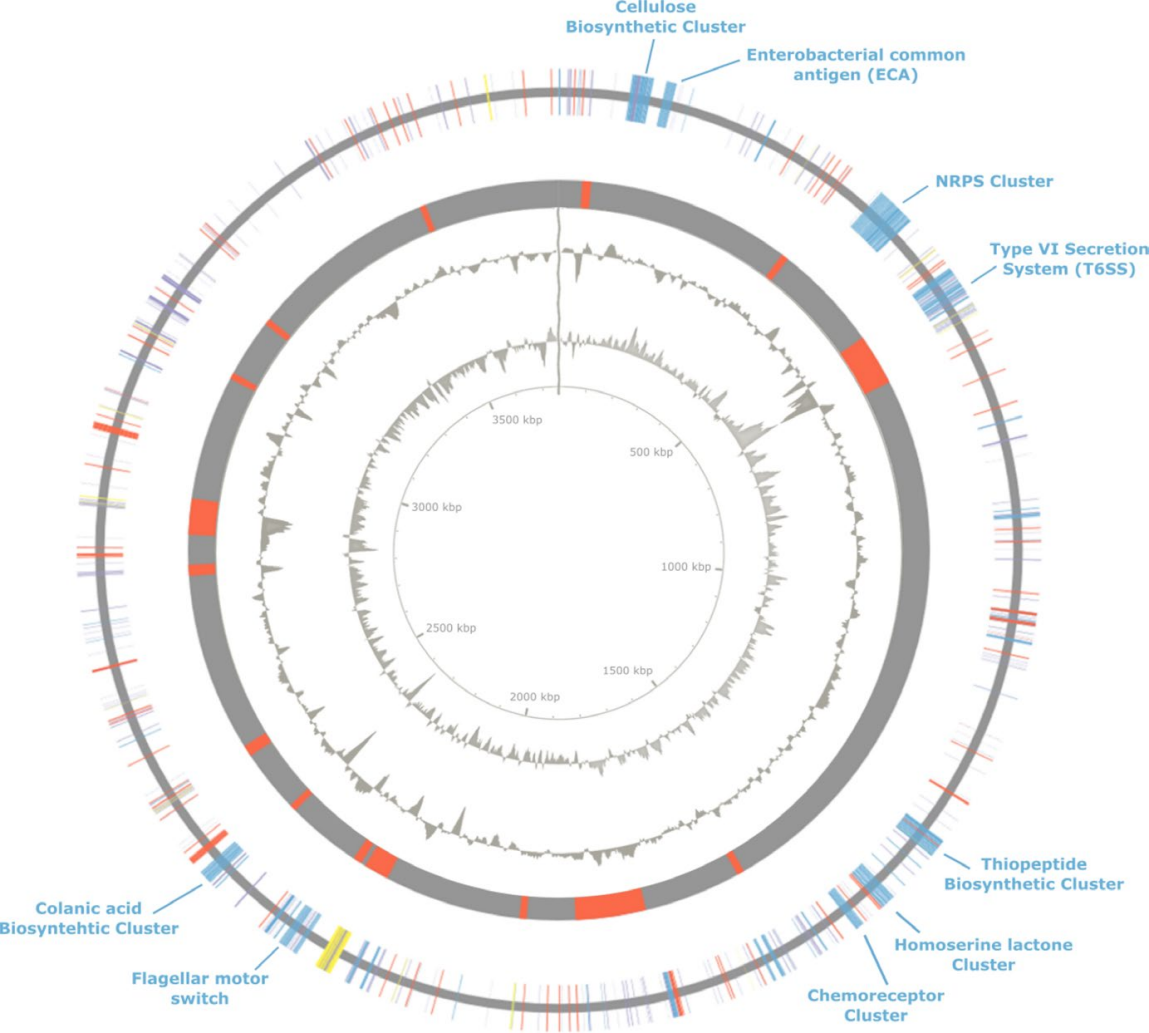
Genome map of the main chromosome of *Pantoea* sp. P37. The outer ring displays specific biosynthetic clusters (blue), MFS transporter, multidrug efflux pumps and transporter (red), hypothetical proteins (purple), and phage‐associated genes (yellow). The second outer ring displays genomic islands (red). The third outer ring represents the GC content. The fourth ring represents the GC skew

**Figure 2:**
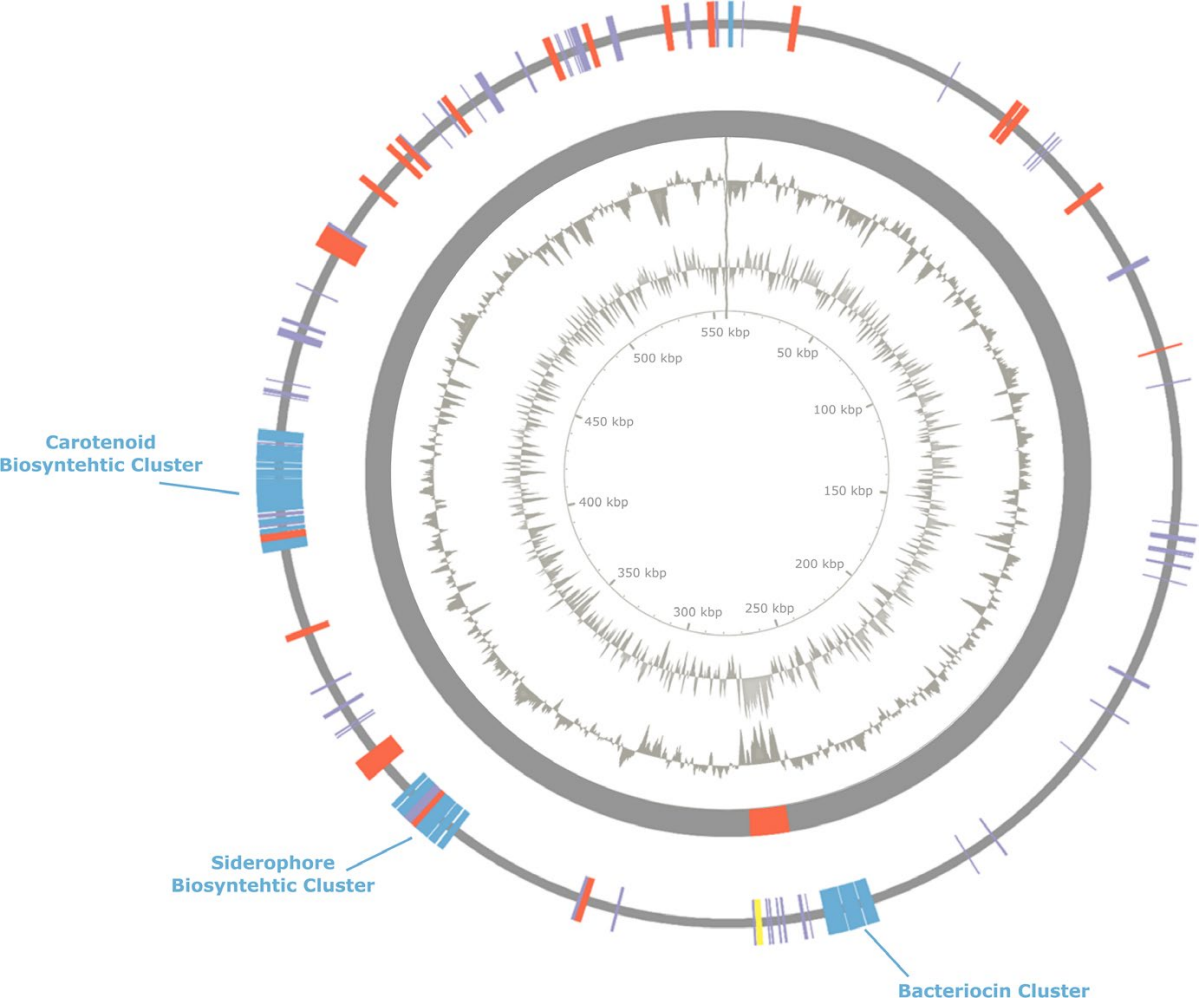
Genome map of *Pantoea* sp. P37 plasmid 1. The outer ring displays specific biosynthetic clusters (blue), MFS transporter, multidrug efflux pumps and transporter (red), hypothetical proteins (purple), and phage‐associated genes (yellow). The second outer ring displays genomic islands (red). The third outer ring shows the GC content. The fourth ring displays the GC skew

**Figure A2:**
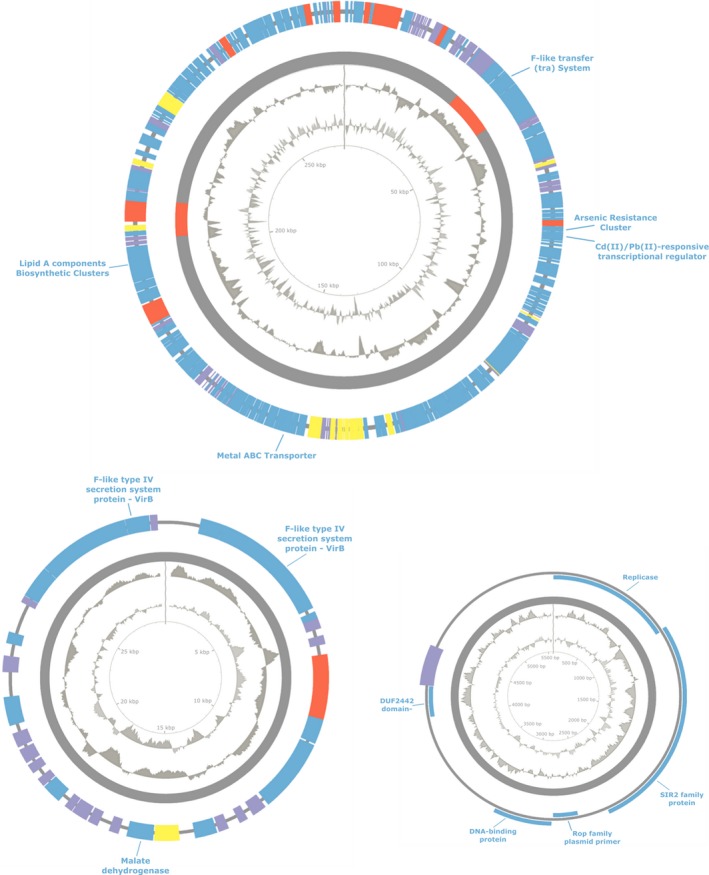
Genome map of the *Pantoea* sp. P37 plasmids 2, 3 and 4. The outer ring displays all known CDS (blue), MFS transporter, multidrug efflux pumps and transporter (red), hypothetical proteins (purple), and phage‐associated genes (yellow). The second outer ring displays genomic islands (red). The third outer ring shows the GC content. The fourth ring displays the GC skew

The genome of *Pantoea* sp. P37 was sequenced and mapped for a deeper analysis of virulence factors. The genome maps highlighting particular features found on the main chromosome of *Pantoea* sp. P37 and its four plasmids are shown in Figures 1, 2, and A2. The *Pantoea* sp. P37 chromosome showed features typical for Enterobacteriaceae and plant‐associated bacteria. Among the identified clusters was the cellulose biosynthetic gene cluster (BGC), the Enterobacterial common antigen cluster (ECA), a nonribosomal peptide synthetase (NRPS), a type VI secretion system (T6SS), flagellae, chemoreceptors, and the colanic acid BGC. In total, 15 genomic islands (GI) were predicted. The majority of phage genes detected were part of the predicted GI. These involved the phage genes encoding the prophage integrases IntA and IntS and the phage shock proteins A, B, C, and D. Other clusters found in the GI region were the surface lipopolysaccharide O‐antigen ligase gene, genes encoding the type VI secretion system tube protein Hcp, the actin cross‐linking toxin VgrG1, and several genes of the flagella cluster. Other genomic elements present in the predicted GIs were stress response‐related genes encoding osmoregulators, SOS response proteins, a p‐hydroxybenzoic acid efflux pump, and general stress proteins. Multiple antibiotic resistances including β‐lactam, virginiamycin, and bicyclomycin resistance were also detected. Carbohydrate metabolism genes occurring in a predicted GI were comprised of three of the four homolog genes required for dTDP‐L‐rhamnose biosynthesis, *rmlA* (*rffH_2*), *rmlC* (*rfbC*), and *rmlD*.

Plasmid 1 carried three BGC which could be identified (Figure 2). One of the clusters contained genetic elements encoding for enzymes participating in bacteriocin synthesis. A siderophore cluster found on plasmid 1 consisted of genes encoding siderophore biosynthesis proteins, an anion permease as well as iron and a major facilitator superfamily (MFS) transporter. An isoprenoid BGC for the production of zeaxanthin was also detected. One GI was predicted in plasmid 1. It was flanked by a phage‐associated gene encoding a transposase and by genes of unknown function. Within the GI, 6 of the 17 CDS were genes of unknown function.

One of the main features of plasmid 2 was a cluster‐like segment composed of various transfer (tra) genes located in one of the two predicted GI (Figure A2). Besides the tra genes on plasmid 2, several genomic sequences were identified which encoded for enzymes involved in the biosynthesis of the endotoxic cell membrane component lipid A. Almost 20% of the plasmid 2 CDS were genes with unknown function.

A range of genes encoding for VirB proteins was also found on plasmid 3 while 43% of all genes had an unknown function. The smallest plasmid 4 with a total size of 5,593 bp contained 5 known and one unknown CDS. No specific BGC or virulence factors could be identified (Figure A2).

### 3.5 Identification and quantification of vector‐dependent mono‐rhamnolipid production

**Figure 3:**
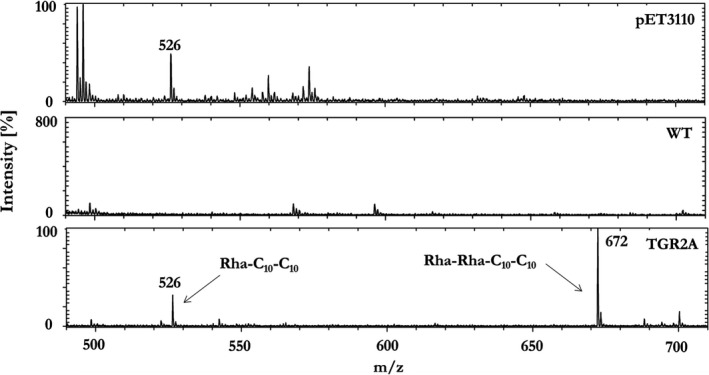
MALDI‐TOF‐MS spectra of extracts from culture supernatants of recombinant *Pantoea* sp. P37 isolate (pET3110), *Pantoea* sp. P37 wild type (WT) and *P. aeruginosa* isolate TGR2A

To confirm the heterologous production of rhamnolipids by *Pantoea* sp. P37, the recombinant products in the culture supernatants were analyzed by Matrix Assisted Laser Desorption Ionization—Time of Flight Mass Spectrometry (MALDI‐TOF‐MS) (Figure 3). A peak at *m/z* 526 represented the major ion of the [M + Na]+adduct of the mono‐rhamnolipid α‐L‐rhamno‐pyranosyl‐β‐hydroxydecanoyl‐β‐hydroxy‐decanoate (Rha‐C_10_‐C_10_). The peak was observed in the culture supernatants of *P. aeruginosa* TGR2A and of the recombinant *Pantoea* sp. P37 strain transformed with pET3110. However, it was not present in the culture supernatant of the wild‐type strain proving the heterologous production of mono‐rhamnolipids by *Pantoea* sp. P37. In addition, a peak at *m/z* 672 indicating the presence of di‐rhamnolipids was only detected in the culture supernatant of the natural rhamnolipid producer *P. aeruginosa* TGR2A. The concentration of the mono‐rhamnolipid Rha‐C_10_‐C_10_ in the culture supernatants of the recombinant *Pantoea* sp. P37 strain transformed with pET3110 and additional vectors was quantified by HPLC (Table 1). The pET3110 transformants produced 409.4 mg/L of the rhamnolipid which was the highest concentration detected. The other transformants produced much lower amounts, and no product was detected in the culture supernatants of the transformants pETrhlAB100 and pET2711.

**Table 1:** Heterologous mono‐rhamnolipid production by *Pantoea* sp. P37 transformed with different vectors

Vector(s)	Entirety of recombinant genes	Mono‐RL [mg/L]
pETrhlAB8	*rhlAB* under BBa_J23108	21.4 ± 1.1
pETrhlAB100	*rhlAB* under BBa_J23100	Not detected
pET3110	*rhlAB* under BBa_J23108 and *rmlBCDA*	409.4 ± 70.8
pET2711	*rhlAB* under BBa_J23100 and *rmlBCDA*	Not detected
pET3110 and pACYC_fabH	*rhlAB, rmlBCDA, fabH*	*2*1.8 ± 1.3
pET3110 and pACYC_P450	*rhlAB, rmlBCDA, fabH2, cytochrome P450*	10.2 ± 8.9
pET3110 and pACYC_FAS	*rhlAB, rmlBCDA, accABCD, acp, fabFDG*	5.4 ± 7.6
pET3110 and pACYC_algC	*rhlAB, rmlBCDA*, *algC*	33.7 ± 4.8

### 3.6 Impact of heterologous rhamnolipid production on the transcriptome of *Pantoea* sp. P37

**Figure 4:**
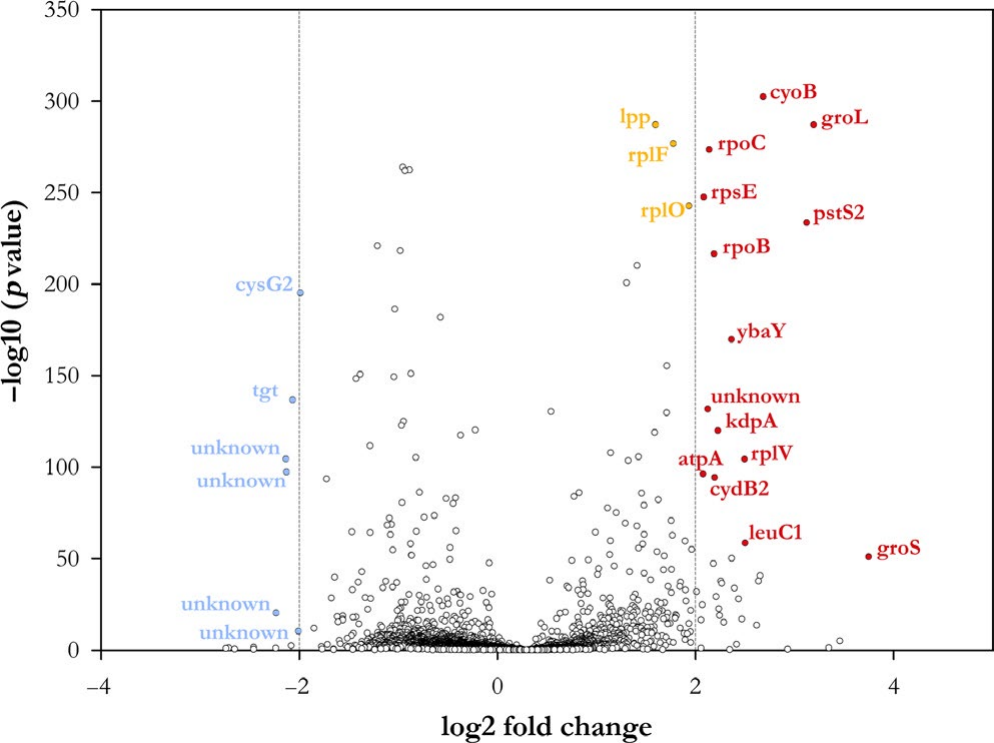
Differential expression analysis of genes expressed in the recombinant *Pantoea* sp. P37 (pETrhlAB8) and its wild type. Blue dots: |log2(FoldChange)| < −2; Red dots: |log2(FoldChange)|> 2; Orange dots: 1.5 <|log2(FoldChange)| < 2. *p*‐Value < .005. *tgt*: Queuine tRNA‐ribosyltransferase; Siroheme synthase 2; *groS*: 10 kDa chaperonin GroES; *leuC1*: 3‐isopropylmalate dehydratase large subunit 1; *cydB2*: cytochrome bd‐I ubiquinol oxidase subunit 2; *atpA*: ATP synthase subunit alpha; *rplV*: 50S ribosomal protein L22; *kdpA*: potassium‐transporting ATPase potassium‐binding subunit; *ybaY*: lipoprotein YbaY; *rpoB*: DNA‐directed RNA polymerase subunit beta; *pstS2*: phosphate‐binding protein PstS 2; *rpsE*: 30S ribosomal protein S5; *rpoC*: DNA‐directed RNA polymerase subunit beta; *groL*: 60 kDa chaperonin GroEL; *cyoB*: cytochrome o ubiquinol oxidase subunit I; lpp: major outer membrane prolipoprotein Lpp; *rplF*: 50S ribosomal protein L6; *rplO*: 50S ribosomal protein L15

An RNA‐seq analysis was performed to assess the impact of the recombinant production of mono‐rhamnolipids on the *Pantoea* sp. P37 transcriptome (Figure 4). The majority of upregulated genes in the recombinant strain are genes encoding proteins involved in genetic information processing pathways including chaperones (*groL*, *groS*), ribosomal proteins (*rpsE*, *rplF*, *rplO*, *rplV*), or polymerases (*rpoB*, *rpoC*). A more than twofold expression was also observed of the genes *cyoB* and *cydB2*, both encoding cytochromes. Additionally, with a log2 fold change value of ~1.6, enhanced expression of the lipoprotein (lpp) gene in the recombinant strain encoding the enterobacterial major outer membrane lipoprotein was found. A significant downregulation of the *cysG2* gene which is involved in heme biosynthesis was observed. However, the majority of down‐ and upregulated genes in the transcriptome of the recombinant strain were unidentified genes.

## 4 DISCUSSION

Screening of soil samples on rhamnolipid‐fortified agar was performed to select microbial strains with resistance to rhamnolipids. As mainly pathogenic members of the ubiquitous *P. aeruginosa* were expected to grow, a colony PCR‐mediated exclusion based on *rhlI*, coding for the acyl‐homoserine‐lactone synthase responsible for activating the expression of the *rhlAB* operon and other virulence‐associated genes was employed (Raychaudhuri, Jerga, & Tipton, 2005). The remaining strains were predominantly identified as pathogenic members of the Enterobacteriaceae family suggesting a genetic preset of resistance to rhamnolipids. A possible mechanism of this bacterial resistance is the production of specific cleaving enzymes or multidrug efflux pumps and transporter proteins of the MFS which provides nonspecific resistance to a number of compounds (Partridge, 2015). *Pantoea* sp. P37 was identified as the only rhamnolipid‐resistant strain with a potential BSL 1.

A prediction of the pathogenicity of novel *Pantoea* strains is problematic and the misidentification of clinical samples has often resulted in the wrong attribution of *Pantoea* species to a variety of infections (Walterson & Stavrinides, 2015). Besides a phylogenetic analysis based on the housekeeping genes 16S rRNA, *gyrB* and *rpoB* (Figure A1), a de novo whole‐genome sequencing was performed to both clarify the identity of the isolate and also to allow an in‐depth pathogenomic evaluation of the strain (Figures 1, 2 and A2; Table A1). The phylogenetic analysis indicated a close relatedness of the isolate to the nonpathogenic strains *P. dispersa* and *P. wallisii*. Also, the structure of its genome, the presence of a range of plant‐associated genes, and the absence of major virulence factors suggest an epiphytic lifestyle of *Pantoea* sp. P37 rather than that of a human pathogen.

A total of four plasmids were present in *Pantoea* sp. P37 which is a common feature of the *Pantoea* genus. The F+‐mediated conjugation system, responsible for horizontal gene transfer, is a major feature of the *Pantoea* genus providing biological robustness by the integration of GI (De Maayer et al., 2012). The GI found in *Pantoea* sp. P37 consisted of genes indicating environmental adaption processes. One of the gene clusters suggesting a plant‐associated lifestyle detected was *tehB* encoding a tellurite methyltransferase and *tehA* encoding a resistance protein against the toxic tellurium dioxide (Chasteen, Fuentes, Tantaleán, & Vásquez, 2009). Other clusters were the carotenoid BGS for protection against UV irradiation and genes supporting the utilization of sucrose (Reid & Abratt, 2005). No genes of the cluster encoding for components of the type III secretion systems (T3SS), an essential prokaryotic virulence determinant of invasive Gram‐negative pathogenic bacteria for injection of effector proteins into plant, animal, or human host cells were found (Deng et al., 2017). *Pantoea* sp. P37 also lacked other virulent determinants including proteases and lipases which target structural components of host cells leading to the dissemination of infection (Titball, 1993). *Pantoea* sp. P37 also did not harbor any major toxin‐encoding sequences similar to *Pantoea vagans* C9‐1, a commercially available biocontrol agent considered as safe (Smits et al., 2010). The only toxin found was the endotoxic surface lipopolysaccharide (LPS) which is part of the outer membrane of all Gram‐negative bacteria, including the safe *E. coli* strain K12 (Brade, 1999). Identified critical clusters like the type VI secretion system (T6SS), chemotactic flagella, or bacteriocin biosynthetic cluster are predominately survival mechanisms used for the ecological adaption to changing environmental processes and interbacterial defense and are considered as safe (Cotter, Ross, & Hill, 2013; Russell, Peterson, & Mougous, 2014; Wadhams & Armitage, 2004). However, the presence of multiple multidrug efflux pumps, MFS domains and the resistance against β‐lactam, virginiamycin, and bicyclomycin needs to be considered.

The heterologous expression of the *rhlAB* operon in *Pantoea* sp. P37 resulted in the production of the mono‐rhamnolipid Rha‐C_10_‐C_10_ with a titer of approximately 0.4 g/L. In contrast, in the early research stage, the recombinant strain of *Pseudomonas putida* KT2440 produced a total rhamnolipid concentration of 0.22 g/L (Wittgens et al., 2011). After fermentation process design experiments for optimized rhamnolipid production, the same strain was able to achieve a titer of 14.9 g/L (Beuker et al., 2016). The highest concentration of mono‐rhamnolipids was obtained by integration of the *rmlBCDA* operon derived from *P. aeruginosa* into the rhamnolipid *rhlAB* expression vector. This is in accordance with a previous study where the availability of dTDP‐L‐rhamnose has been considered as a limiting factor for in the recombinant production of mono‐rhamnolipids in *E. coli* (Cabrera‐Valladares et al., 2006). The dTDP‐L‐rhamnose limitation may be due to the presence of several pathways competing for carbohydrates which are common in Enterobacteriaceae but not in *Pseudomonads*.

The relatively low production of mono‐rhamnolipids following the integration of the *rmlBCDA* operon derived from *P. aeruginosa* could suggest that the lipid precursor‐generating pathway for the rhamnolipid synthesis has been a limiting factor. Many studies have focused on the identification of the key pathway providing the lipid precursor for rhamnolipid synthesis, however, with ambiguous conclusions. While some studies favoured a de novo fatty acid synthesis (Gutiérrez‐Gómez, Servín‐González, & Soberón‐Chávez, 2019), others concluded that the β‐oxidation pathway is the main contributor (Abdel‐Mawgoud, Lépine, & Déziel, 2014) or that both pathways are contributing precursors (Zhang, Veres‐Schalnat, Somogyi, Pemberton, & Maier, 2012). In this study, the coexpression of several vectors carrying genes involved in the de novo fatty acid biosynthesis and possibly in the formation of the lipid moiety of the rhamnolipids, such as the *accABCD* and *fabFDG* genes, was compared. However, none of the fatty acid vectors did improve the production of mono‐rhamnolipids. Additional experiments dealing with the optimization of protein expression levels are required to investigate the effects on the level of rhamnolipids produced.

The transcriptome analysis revealed a significant upregulation of the genes *cyoB*, *groL,* and *groS* in *Pantoea* sp. P37 during recombinant rhamnolipid production. The enhanced expression of the cytochrome o ubiquinol oxidase subunit I encoding gene *cyoB* indicated changes in the respiratory system of the cells due to redox imbalances (Yap et al., 2010). The enhanced expression of genes of the chaperonins GroEL and GroES likely represents natural prokaryotic stress responses (Kumar, Mande, & Mahajan, 2015). These results suggest that the heterologous production of rhamnolipids induced stress to the cells which reduced the fitness of *Pantoea* sp. P37 explaining its inability to produce mono‐rhamnolipids when *rhlAB* expression was driven by a strong promoter.

Surfactants adversely affect cell wall membranes by phospholipid solubilization thereby increasing the permeability of the membrane (Otzen, 2017). The lpp gene of gamma‐proteobacteria plays a key role in maintaining homeostasis and structural integrity of the cell wall. Increased expression of the lpp gene, a major outer membrane lipoprotein in the *Pantoea* sp. P37 mutant carrying the *rhlAB* expression vector was observed indicating that in the presence of rhamnolipids, the cells were forced to overexpress the lpp gene to stabilize the cell wall membrane.

The aim of this work was to enable the biosynthesis of rhamnolipids in a nonpathogenic and recombinogenic bacterial strain as an alternative to the opportunistic pathogen *P. aeruginosa*. *Pantoea* sp. P37 could be isolated as a potential BSL 1 host which allowed the application of cloning methods, expression vectors, and promoter sequences previously developed for *E. coli*. However, the achieved rhamnolipid concentrations were low compared to *P. putida* KT2440. As shown by the transcriptome analysis, this could be caused by insufficient precursor molecules and by the inherent toxicity of the produced rhamnolipids and its detrimental effects on the host. Further development of this strain should focus on increasing the supply of precursors for rhamnolipid biosynthesis by metabolic engineering, further increasing the resistance of the host against rhamnolipids, that is, by adaptive evolution, the removal of undesired genes and the design of a fermentation process for optimized rhamnolipid production.

## CONFLICT OF INTEREST

None declared.

## AUTHOR CONTRIBUTIONS

Margarete Nawrath conceived the study, curated the data, performed formal analysis, involved in the investigation, contributed to the methodology and visualization, and wrote the original draft. Christoph Ottenheim conceived the study, curated the data, performed formal analysis, involved in supervision and validation, and wrote, reviewed, and edited the manuscript. Jinchuan Wu conceived the study; acquired fund; involved in the investigation, project administration, resources, and supervision; and wrote, reviewed, and edited manuscript. Wolfgang Zimmermann conceived the study, contributed to the methodology, supporting, and supervision; wrote, reviewed, and edited the manuscript.

## ETHICS STATEMENT

None required.

## Data Availability

The genome sequencing data of *Pantoea* sp. P37 were submitted to the NCBI Sequence Read Archive under accession numbers https://www.ncbi.nlm.nih.gov/sra/SRR7962492 and https://www.ncbi.nlm.nih.gov/sra/SRR7962491. Its 16S rRNA sequence was deposited under accession number https://www.ncbi.nlm.nih.gov/nuccore/MH071150 at NCBI. The *Pantoea* sp. P37 genome was deposited in the NCBI repository under accession number https://www.ncbi.nlm.nih.gov/nuccore/CP032702‐https://www.ncbi.nlm.nih.gov/nuccore/CP032706, BioProject ID https://www.ncbi.nlm.nih.gov/bioproject/PRJNA494309/, biosample ID https://www.ncbi.nlm.nih.gov/biosample/SAMN10160529/.

## Appendix 1

**Table A1:** Features of the genome of *Pantoea* sp. P37

Feature	Chromosome	Plasmid 1	Plasmid 2	Plasmid 3	Plasmid 4
Size (bp)	3,758,528	556,856	275,665	29,830	5,593
G + C content	57.8%	57.9%	52.8%	45.5%	47.5%
ORFs (≥100 bp)	36,916	5,806	2,678	269	57
CDS	3,384	517	252	37	6
Genes of known function	3,105	456	204	21	5
Hypothetical proteins	279	61	48	16	1
ncRNAs	8	0	0	0	0
rRNAs	22	0	0	0	0
tRNAs	80	0	0	0	0

**Table A2:** Genome assemblies from Pantoea strains downloaded from NCBI

Strain designation	NCBI accession number
*P. agglomerans* C410P1	CP016889.1
*P. vagans* C9‐1	CP002206.1
*P. brenneri* LMG 5343	GCA_002095305.1
*P. ananatis* LMG20103	CP001875.2
*P. stewartii* subsp. *stewartii* DC283	CP017581.1
*P. septica* FF5	GCA_000612605.1
*P. rodasii* LMG26273	GCA_002095465.1
*P. rwandensis* ND04	CP009454.1
*P. eucrina* LMG5346	GCA_002095385.1
*P. wallisii* LMG26277	GCA_002095485.1
*P. dispersa* EGD‐AAK13	GCA_000465555.2
*P. carbekii*	NC_022547.1
*E. coli* K12	NC_000913.3

**Table A3:** Overview of the oligonucleotides used and their respective nucleotide sequences

Name	Sequence
27F	5'‐ AGAGTTTGATCMTGGCTCAG‐3'
1492R	5'‐CGGTTACCTTGTTACGACTT‐3'
rhlI_F	5'‐GTTTGCGGATGGTCGAACTG‐3'
rhlI_R	5'‐AGAGGGCCCAGGAGTATCAG‐3'
pET_SF	5'‐GGGGAAATGTGCGCGGAAC‐3'
pET_LF	5'‐CAGGTGGCACTTTTCGGGG‐3'
pET_SR	5'‐CAGAATGAATCACCGATACGCGAG‐3'
pET_LR	5'‐GTTGCCTTACTGGTTAGCAGAATGAATC‐3'
J23108_F	5'‐CTAACCAGTAAGGCAACCCTGACAGCTAGCTCAGTCCTAGGTATAATGCTAGC‐3'
J23108_R	5'‐CTCCTTCTTGTCTCTTAGCTAGCATTATACCTAGGACTGAGCTAGCTGTCAG‐3'
rhlA_LF	5'‐CTAAGAGACAAGAAGGAGGTTAGTTTATGCGGCGCGAAAGTC‐3'
rhlA_SF	5'‐GTTAGTTTATGCGGCGCGAAAGTCTG‐3'
rhlA_OER	5'‐AATTAGGTTATCTTAGGAGGGGCCATCAGGCGTAGCCGATGG‐3'
rhlB_OEF	5'‐TGGCCCCTCCTAAGATAACCTAATTATGCACGCCATCCTCATCG‐3'
rhlB_SR	5'‐GCGAAAAAACCCCGCCGAAGCGGGGTTTTTTGCGTCAGGACGCAGCCTTCAG‐3'
rhlB_LR	5'‐GAAAAGTGCCACCTGGCGAAAAAACCCCGCCG‐3'
rml_SR	5'‐TCAGGGGAAGCAGTCGGCG‐3'
rml_LR	5'‐CAACATGAATGGTCTTCTCAGGGGAAGCAGTCGGCG‐3'
rml_SF	5'‐TGAAGGAAGAGAGGTAATATATGACGATTCTCGT‐3'
rml_LF	5'‐CCTAGGTATAATGCTAGCTGAAGGAAGAGAGGTAATATATGACGATTCTCGTGACCGGC‐3'
rhlvb_SF	5'‐TTGCTCAGGTCGCAGACGT‐3'
rhlvb_LF	5'‐GAAGACCATTCATGTTGTTGCTCAGGTCGCAGACGT‐3'
rhlvb_SR	5'‐ACTGAGCTAGCTGTCAATATCAGCTC‐3'
rhlvb_LR	5'‐GCTAGCATTATACCTAGGACTGAGCTAGCTGTCAATATCAGCTCACTCAAAGGCGGTAAT‐3'
pACYC_LF	5'‐GGCATTTGAGAAGCACACGGTCACACTGCTTCCG‐3'
pACYC_SR	5'‐GACTGAGCTAGCCGTAAACGTATGG‐3'
pACYC_LR	5'‐GCTAGCACTATACCTAGGACTGAGCTAGCCGTAAACGTATGGGGCTGACTTCAGGT‐3'
P450_SF	5'‐TAAGGAAAGGGTAGTATAGTGCCTGATCGCAAACTGAGAC‐3'
P450_LF	5'‐CTAGGTATAGTGCTAGCTAAGGAAAGGGTAGTATAGTGCCTGATCG‐3'
fabH2_SR	5'‐TCAGTCCATTGTCGGAACGATCTTC‐3'
fabH2_LR	5'‐TGCTTCTCAAATGCCTCAGTCCATTGTCGGAACGATCTTC‐3'
fabH22_SF	5'‐CAATCGGAGGAGGCCTAAGCCATGCCGCGCG‐3'
fabH22_LF	5'‐CTAGGTATAGTGCTAGCCAATCGGAGGAGGCCTAAGC‐3'
fabDFG_SF	5'‐CTGAAAGGAGGTATTTTTTTATGTCTGCATCCCTCGCATTC‐3'
fabDFG_LF	5'‐CTAGGTATAGTGCTAGCCTGAAAGGAGGTATTTTTTTATGTCTGCATCCCTCGCATTC‐3'
fabDFG_SR	5'‐TAGGACTGAGCTAGCTGTCAATCAGTCGGCGAACCTGCG‐3'
fabDFG_LR	5'‐GGCTAGCACAATACCTAGGACTGAGCTAGCTGTCAATCAGTCGGCGAACCTGCG‐3'
accA_SF	5'‐CCTAGGGGAGACGTCTTTAATGAACCCGAACTTTCTTGATTTCGA‐3'
accA_LF	5'‐GGTATTGTGCTAGCCCCTAGGGGAGACGTCTTTAATGAACCCGAACTTTCTTGATTTCGA‐3'
accA_SR	5'‐AATTACGGCGCGCCGTAGCT‐3'
accA_LR	5'‐GGGTAAACTCCTTCTCAATTACGGCGCGCCGTAGCT‐3'
accBC_SF	5'‐ATATGGACATTCGTAAAGTCAAGAAACTGATC‐3'
accBC_LF	5'‐GAGAAGGAGTTTACCCATATGGACATTCGTAAAGTCAAGAAACTGATC‐3'
accBC_SR	5'‐CAGTGCTTGTCCATACCCAGTTTCTTC‐3'
accBC_LR	5'‐ATATACCTCCTTTTCTGTCAGTGCTTGTCCATACCCAGTTTCTTC‐3'
accD_SF	5'‐TACATGAGCAACTGGCTGGTAGACAAG‐3'
accD_LF	5'‐ACAGAAAAGGAGGTATATTACATGAGCAACTGGCTGGTAGACAAG‐3'
accD_SR	5'‐GTGACCGTGTCATGCGGATACGGGGCTC‐3'
accD_LR	5'‐CTACCGGAAGCAGTGTGACCGTGTCATGCGGATACGGGGCTC‐3'
